# Detailed molecular epidemiology of *Chlamydia trachomatis* in the population of Southampton attending the genitourinary medicine clinic in 2012-13 reveals the presence of long established genotypes and transitory sexual networks

**DOI:** 10.1371/journal.pone.0185059

**Published:** 2017-09-25

**Authors:** Clare Labiran, David Rowen, Ian Nicholas Clarke, Peter Marsh

**Affiliations:** 1 Molecular Microbiology, Clinical and Experimental Sciences, Faculty of Medicine, University of Southampton, Southampton, United Kingdom; 2 Department of GU Medicine, Royal South Hants Hospital, Southampton, United Kingdom; 3 Public Health England, Public Health Laboratory Southampton, Southampton General Hospital, Southampton, United Kingdom; Universita degli Studi di Bologna, ITALY

## Abstract

*Chlamydia trachomatis* is the most common sexually transmitted infection (STI) in England. Our objective was to perform a detailed survey of the molecular epidemiology of *C*. *trachomatis* in the population of Southampton UK attending the genitourinary medicine clinic (GUM) to seek evidence of sexual network activity. Our hypothesis was that certain genotypes can be associated with specific demographic determinants. 380 positive samples were collected from 375 *C*. *trachomatis* positive GUM attendees out of the 3118 who consented to be part of the survey. 302 of the positive samples were fully genotyped. All six of the predominant genotypes possessed *omp*A locus type E. One ward of Southampton known to contain a large proportion of students had a different profile of genotypes compared to other areas of the city. Some genotypes appeared embedded in the city population whilst others appeared transient. Predominant circulating genotypes remain stable within a city population whereas others are sporadic. Sexual networks could be inferred but not conclusively identified using the data from this survey.

## Introduction

The greatest impact of sexually transmitted infections (STIs) in England is among young heterosexuals under the age of 25, and men who have sex with men (MSM). The most common sexually transmitted infection (STI) in England is caused by *Chlamydia trachomatis*, responsible for the highest rates with 200,288 diagnoses in 2015 [1]. Teenage conception rates remain high in England, and in 2012 the rate in Southampton females under sixteen years of age was among the top five cities in the South East of England[2]. The majority of infections occur within sexual transmission networks as illustrated by a study of clonal relationships between *C*. *trachomatis* samples from an STI clinic in Amsterdam [3]. There is a need to identify sexual networks to understand how strains move through a population, and to determine whether certain strains are moving within particular networks. Strain characteristics such as tropism may be selectable in specific sexual networks; selective advantage may enable accelerated proliferation of a significant new emerging strain. Strains move between networks via bridging populations (e.g. between MSM and heterosexual networks via bisexuals)[3]. Genotyping of *Neisseria gonorrhoeae* has been used to define patient groups with similar demographic characteristics [4]. Clones may emerge within particular networks with selectable factors such as virulence, recalcitrance to treatment or detection evasion such as the Swedish new variant *C*. *trachomatis* (nvCT) [5]. Sexual networks are heterogeneous, and as in other social networks through which infectious diseases spread, a relatively small number of individuals within that network are usually responsible for a large proportion of the infections, the so called “super-spreader” phenomenon. However, unlike diseases which rely on relatively simple and low levels of contact (for example influenza), sexual networks are complicated by the number and nature of sexual partners an individual has, and the frequency and risk factor of sex acts with each partner (which may differ between partners) [6]. High resolution genotyping may be used to define predominant clones within geographic areas and within demographic groups. Temporal differences are also important to observe: some genotypes might be “endemic” in a population whereas others might occur in short temporal bursts suggesting mini-outbreaks or invasion/occurrence of a new genotype which does not establish.

Clinical, biological and epidemiological discrimination of strains is the ultimate goal of an ideal genotyping method. Whole genome sequencing (WGS) achieves this, but especially in the case of *C*. *trachomatis* (which often requires isolation in cell culture to obtain complete genome sequences), it is not currently practicable to use for all samples one might collect in a meaningful survey. Genotyping systems which target multiple *loci* remain powerful tools for molecular epidemiology, as demonstrated in several recent studies[3, 7–12]. Two key components in a molecular epidemiological study of a city population are high quality prospectively collected sample material, and meaningful demographic data. There have been several significant city-population studies using multi locus sequence typing (MLST) methods[3, 13]. Pedersen et al (2008)[7] developed a high resolution genotyping system based upon multi-locus variable number tandem repeat (VNTR) analysis plus analysis of the *omp*A gene (MLVA-*omp*A), the latter being the locus originally used in *C*. *trachomatis* genotyping[14, 15]. This was successfully validated in an evaluation in which swabs from Southampton women were taken in 2009[16]. Recent work showed that these VNTR marker sequences are stable, therefore suitable for genotyping[17]. The technique PCR-amplifies four short markers (three VNTR *loci* and the *omp*A gene), followed by DNA sequence analysis, and optimally differentiates between genotypes[18] as measured by the Simpson’s discriminatory index[7, 16]. It has recently revealed evidence of a HIV-related sexual network in men who have sex with men (MSM) in Brighton, UK[12], and a Japanese study provided evidence of how this scheme finely discriminates between distinct genotypes within the *omp*A types[19].

Our aim was to conduct a detailed molecular epidemiological study of *C*. *trachomatis* in the genitourinary medicine clinic (GUM)–attending Southampton population and to investigate whether it was possible to obtain evidence of sexual networks. The existence of prior data from our previous study also enabled us to make observations linking to two sets of data. [16], We hypothesise that certain genotypes are linked to particular demographic features, and that certain genotypes are embedded in a population whilst others are transient.

## Materials and methods

### Participants

Female and male participants 16 years of age or older attending the Southampton GUM Clinic (Royal South Hants Hospital Sexual Health) were recruited from September 2012 to April 2013. Written consent was obtained prior to collection of routine specimens.

### Specimen collection and storage

Patients’ samples (urine or occasionally urethral or pharyngeal swabs from males; and cervical, or vulvo-vaginal urine specimens from females) were collected together with linked demographic and clinical data (according to the ethical guidance). These were sent to the Health Protection Agency (now Public Health England) Molecular Diagnostics Unit at Southampton General Hospital for routine analysis of *C*. *trachomatis* using the RealTime CT/NG assay (Abbott Molecular, Maidenhead, UK). Following routine analysis and reporting of the results to the GUM clinic, the samples were held at 4°C for one week by the Molecular Diagnostics Unit before the *C*. *trachomatis* positive samples were released for further analysis in the present study. At this point, the samples (in the multi-Collect Specimen Collection Kit transport buffer, Abbott Molecular, Maidenhead, UK) were transferred to a sterile 2 mL screw top tube and labelled with a study number untraceable to the original sample, allowing unlinked anonymization (only demographic data, recorded on a Microsoft Excel spreadsheet remained linked to the study number). These anonymized samples were transferred to the University of Southampton Molecular Microbiology Group and stored at -20°C until genotyping could be performed.

### Patient demographic and clinical data

Demographic information collected and linked to each sample included date of sample collection, sex, age, occupation, ethnicity, sexual orientation, the postal district (first four digits of the postcode) of their home address, and partner positivity if declared. Clinical data included presence or absence of symptoms, and if the former: whether these included dysuria, discharge, odd sensation or irritation in the urethra with testicular or epididymal pain in males, and in females irregular bleeding, menorrhagia, dyspareunia/pelvic pain and increased vaginal discharge.

### DNA sequence analysis of MLVA-*ompA* markers

DNA extraction from the NAATs swab and urine specimens, PCR amplification of VNTR and *omp*A sequences, and sequence analysis and assignment of MLVA-*omp*A genotype designations[7] was carried out as described in Labiran et al (2016)[12]. VNTR and *omp*A sequences were amplified using primers for the VNTR *loci* CT1335, CT1299, CT1291 and for the *omp*A gene according to Labiran et al (2016)[12]. Amplicons from the four PCR reactions per sample (1ng/μl/100 bp) were sent for sequencing by Source Bioscience (Nottingham, UK)[12]. Each VNTR *locus* was compared to the sequences described by Pedersen et al (2008)[7] and Wang et al (2011)[16]. Each VNTR *locus* was given a single numeric designation (1, 2, 3, or 4, *etc*) according to its nucleic acid signature, resulting in a three-digit MLVA designation for the sample corresponding to the *loci* in the order: CT1335, CT1299 and CT1291. Each nucleotide difference at each *locus* leads to a unique single numeric designation. For the fourth sequence, the *omp*A gene, sequence data for each sample was compared to the NCBI database[20] and *omp*A sequence type was assigned accordingly: the alphabetical *omp*A genotype was then added to the three-digit MLVA type to give the MLVA-*omp*A designation (e.g. 8.5.1-E).

Cluster analysis of genotypes was by construction of minimum spanning trees (MSpT) using Bionumerics 7 (Applied Maths, NV, Belgium), which represents genotypes according to the four-*locus* identity[12]. Clusters that differ by no more than one of the four *loci* are linked by a black line. The founder of the MSpT was defined as the cluster with the most single-locus variants (SLVs). Secondary founders were defined as centred on clusters which have the second most SLVs. Sub-groups were defined as consisting of clusters which were at least two SLVs from the nearest founder or sub-founder.

### Statistical methods

The Pearson’s Chi squared test or the Fisher’s exact test (using the statistical software package SPSS version X21) were used where applicable for statistical analyses of associations between CT clusters and clinical parameters. A P value of less than or equal to 0.05 was considered significant. The discriminatory power (D: the typing system’s probability that it will identify a different genotype to two unrelated samples in a population) of the MLVA-*omp*A typing system was calculated using the Simpsons index of diversity[21].

### Ethics

Ethical approval for this study was given by the National Research Ethics Committee (NREC) (Reference number -12/LO/102, study title: Study on the epidemiology of Chlamydia in Southampton).

## Results

### Participants

During the period of September 2012 to April 2013, 3118 individuals (1653 female and 1465 male) consented to participate. 380 positive samples were collected from 375 patients comprising 184 males and 191 females. Five individuals gave two samples each on day of sampling. One (male) had the same MLVA-*omp*A genotype in both urine samples (8.6.1-E). The other four (two females and two males) had different MLVA-*omp*A genotypes in their paired samples.

### Genotypes identified

**Fig 1:**
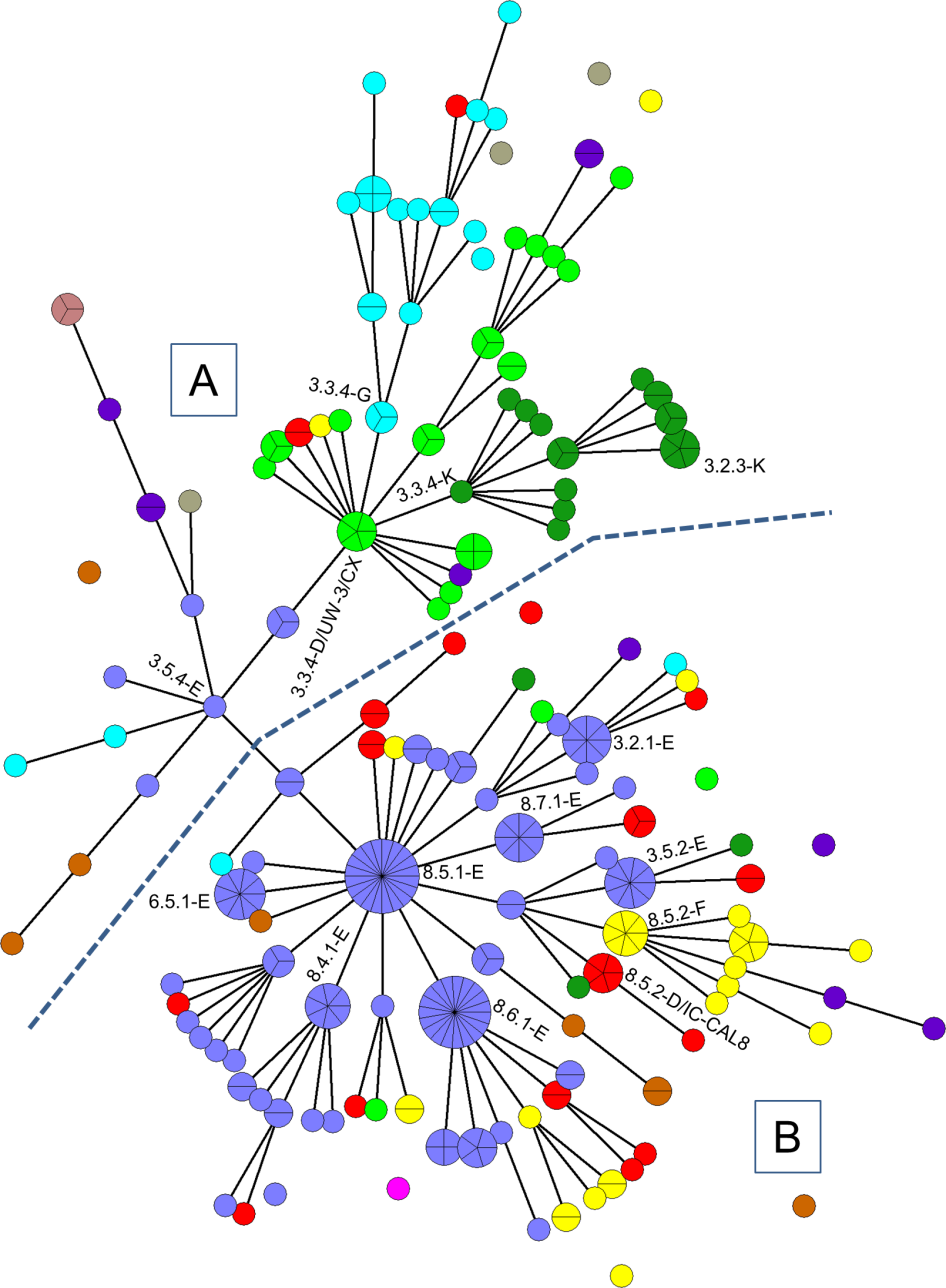
Minimum-spanning tree of 302 samples from Southampton. Each coloured circle represents a MLVA-ompA genotype. Segmentation within the circles shows the number of samples per genotype, branches show single-locus variants (SLV). Unlinked circles show genotypes which differ from nearest linked genotype by more than a SLV. Hand-drawn blue line delineates user-defined sub-groups based on sub-founder groups A and B. The circle colour coding shows ompA genotypes as follows: grey = B; green = D (subtype D/UW-3/CX); red = D (subtype D/IC-Cal8); lavender = E; yellow = F; blue = G; pink = H; brown = I; purple = J; dark green = K. Prominent MLVA-*omp*A genotypes are labelled.

Full four-digit MLVA-*omp*A genotype designations (*i*.*e*. where full sequence data was obtained for all four *loci*) were obtained for 302/380 (79.5%) of the collected positives. For this set of results, the MLVA-*omp*A genotyping scheme gave a discriminatory power (D)of 0.98[21]. Four new VNTR variants for *locus* CT1299 and two new variants for *locus* CT1335 were identified in this study, and numbered as follows. For CT1299: new variant 10 which consisted of fifteen C residues at this *locus* (15C); new variant 11 (16C); new variant 12 (18C); and new variant 13 (21C). For CT1335: new variant 14 which consisted of fourteen T residues and seven A residues at this *locus* (14T7A); new variant 15 (11T9A)[7, 16]. Amongst the fully genotyped samples in this study there were eleven *omp*A genotypes (S1 Table). The four most prevalent *omp*A genotypes were (in descending order): E (45%), D (20%; comprising D/UW-3 at 11% and D/IC-CAL8 at 9%), F (10%) and G (8%), and 149 unique MLVA-*omp*A types were found (S1 Table). *Omp*A genotype D is differentiated into two distinct subtypes because in genomic analysis D/UW-3 and D/IC-CAL8 cluster to distinct clades[22]. There were 58 MLVA (three-digit) signatures unique to a single *omp*A genotype (*e*.*g*. 6.5.1 was only associated with *omp*A genotype E), whereas there were 38 MLVA signatures associated with more than one *omp*A genotype (usually two to four). One MLVA signature (3.3.4) was associated with seven different *omp*A genotypes (D/UW-3, D/IC-CAL8, E, F, G, J and K; S1 Table). The six most prevalent MLVA-*omp*A genotypes were 8.5.1-E (20/302), 8.6.1-E (19/302), 3.5.2-E and 6.5.1-E (both 9/302), and 3.2.1-E and 8.7.1-E (both 8/302) (Fig 1). Three samples (from patients: white British; Asian and unknown) had the *omp*A B3/IU-FQ279 (henceforth referred to as B) locus. Eight samples (from white British patients) had the *omp*A Ia locus. One sample (male heterosexual with symptoms) had the *omp*A L2b locus.

### Cluster analysis of genotype distribution

Among the 302 fully genotyped samples, 147 unique MLVA-*omp*A sequence types were found. Based on single locus variants (SLVs), 290 samples were clustered based on the four loci in the typing scheme and formed one large network representative of the population that attended the GUM clinic in Southampton. The remaining twelve samples had more than a single locus difference to the samples in this network and are shown as unconnected circles in the MSpT (Fig 1). The primary founder cluster of the MSpT was defined as the MLVA-*omp*A genotype with the most SLVs[23], which was MLVA-*omp*A type 8.5.1–E (20 specimens: Fig 1). There were other clusters with a high number of SLVs (≥ six) which could constitute sub-founder groups, such as 8.6.1-E (which was the second largest cluster: 19 specimens), 3.3.4-D/UW-3/CX, 3.3.4-K and 3.5.4-E. *Omp*A genotypes E, D/UW-3CX, G and K were relatively closely clustered within the MSpT, whereas *omp*A genotypes D/IC-CAL8 and F were more dispersed (Fig 1). To analyse the MSpT statistically, although one complete network of SLV-connected clusters occurred, Fig 1 was dividable into two distinct sub-groups, namely A and B, separated by the blue dashed line. Sub-group A consisted of largely *omp*A genotypes D/UW-3CX, G, H, J and K, whereas sub-group B consisted largely of D/IC-CAL8, E and F (P = <0.001).

### Demographic and clinical associations

**Fig 2:**
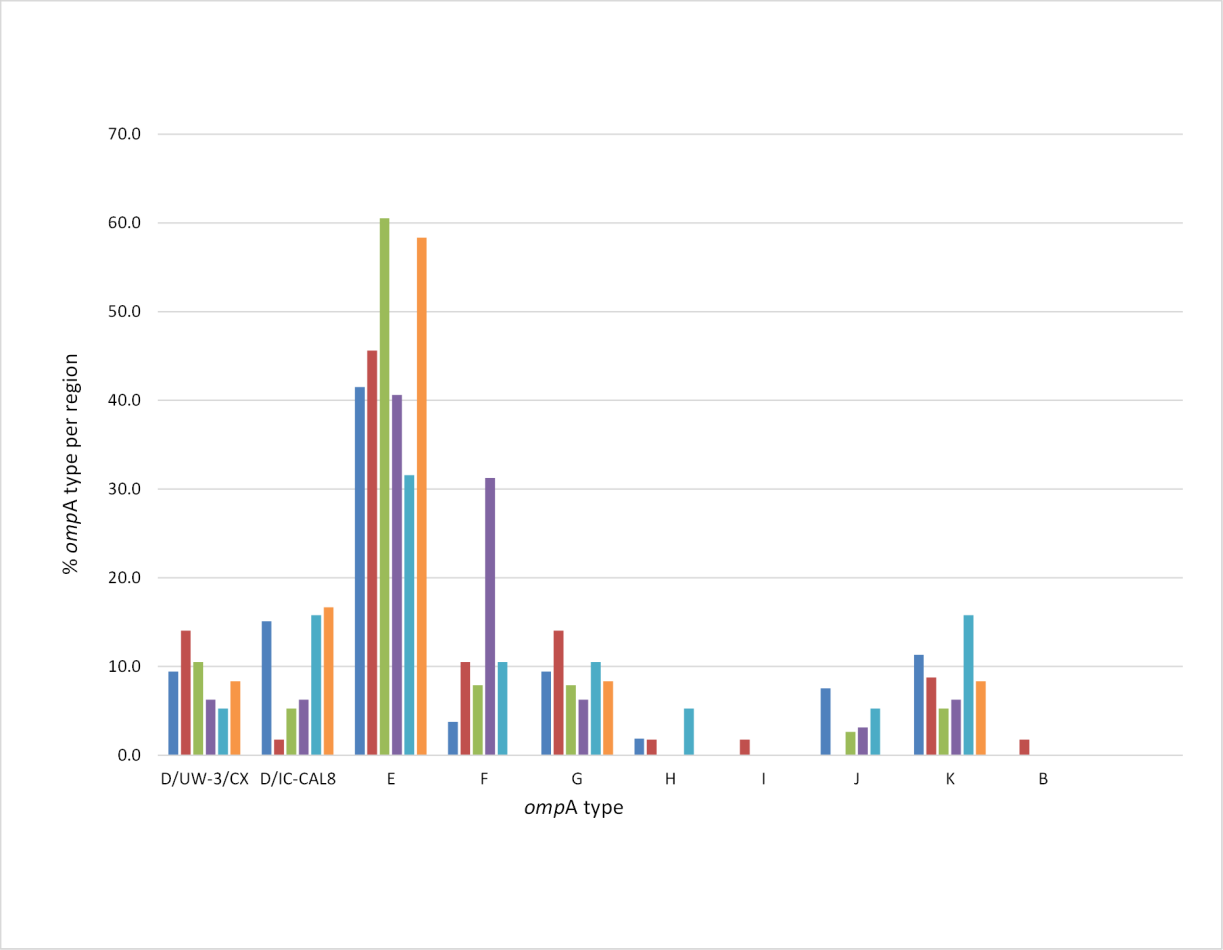
Distribution of *omp*A genotypes (as a percentage of the total positives for the city, n = 223) in regions of the city of Southampton. Colour coding shows Southampton wards or ward-groups represented by four-digit postcodes: blue = SO14; red = SO15; green = SO16; purple = SO17; light blue = SO18; and orange = SO19.

The age range of participants who had positive chlamydia samples was 16–79 for female participants and 17–60 for male participants (the age distribution of the total specimens received was 16–79 for females and 16–86 for males). The mean age was 24, with a median age of 21. The positivity rate was higher in individuals between the ages of 16–24 years old, whilst there was a higher percentage of individuals who were 25 years and above who were negative for *C*. *trachomatis* (p<0.001). There was no significant association between ethnicity or clinical observations, and clusters of genotypes. No symptoms were recorded in 57.6% of the female positives or 46.8% of the male positives. There was no significant association between symptoms or lack of symptoms, and clusters of genotypes. The majority of the positives were from heterosexuals (278/302), whereas the remaining 24 specimens were from MSM (12/302) and orientation-unknown (12/302). Of the twelve MSM, 7/12 were *omp*A genotype D/IC-CAL8 two were J two were E and one, G (S2 Table). The most common ethnic group of the genotyped positives were white British (227/302), with white other (22/302) and black (African, Caribbean or other; 18/302) comprising the next most numerous ethnic groups. Most of the participants were from Southampton and the surrounding areas. However, there were some participants who gave postcodes from far afield such as Cumbria, Lancashire and Stafford. Of the 302 genotyped positive specimens, 223/302 arose from within Southampton City. The four-digit postcodes of participants allowed discrimination of following six regions comprising wards or groups of wards as follows: SO14; SO15; SO16; SO17; SO18; SO19 (Fig 2). Five genotype 8.5.1-E were sampled from SO16, whereas only one specimen was recovered from SO17, and the second most common genotype (8.6.1-E) was found in four regions (SO14, SO15, SO16 and SO19). The percentage of *omp*A genotype F ranged from 0 to 11%, in all the regions apart from SO17, whereas in SO17 F constituted 31% of the genotypes (Fig 2). In SO16, genotype E was present in 61% of the positives; in SO17, 41% and in SO18, 32% (Fig 2). Six specimens that were genotype 8.5.1-E were collected from adjacent regions in Southampton (SO15, SO16, SO17 and SO18) between the 19^th^ and 27^th^ March 2013 (three males, three females; five white British, one unknown) (S3 Table).

### Comparison of genotypes detected in present study to genotypes detected in Southampton in 2009

We previously conducted an evaluation of the MLVA-*omp*A genotyping scheme[7] by collecting swabs from Southampton women and analysing direct DNA extracts as well as cultured isolates were possible[16]. Although no demographic data were collected for that evaluation, and only swabs and hence women’s samples were collected (because during the period of collection all men’s samples were urine), the results from the present study never the less afforded an opportunity to compare genotype coverage in the same city in 2009[16] and 2012–13 (present study) (S1 and S2 Figs). There were 125 genotypes which were detected in 2013 only. Thirteen of the unique-to-2013 genotypes were represented by three or more samples (Table 1 and S4 Table).

**Table 1:** MLVA-*omp*A genotypes represented by three or more samples unique to 2012–13.

MLVA-*omp*A genotype	Sex (number MSM if M)	Number of samples	Period Collected
3.5.2-E	F	6	Dec 2012-Mar 2013
	M (1)	3	
8.6.4-E	F	1	Nov 2012-Jan 2013
	M (0)	3	
3.2.1-E	F	0	Sep 2012-Oct2012
	M (1)	8	
8.7.1-D/IC-CAL8	F	1	Oct 2012-Feb 2013
	M (2)	2	
3.6.1-E	F	4	Mar 2013-Apr 2013
	M (0)	1	
3.3.4-D/UW-3/CX	F	0	Oct 2012-Nov 2012
	M (0)	5	
3.2.3-K	F	3	Oct 2013-Jan 2013
	M (1)	2	
8.3.4-D/UW-3/CX	F	1	Nov 2012
	M (0)	2	

F: Female

M: Male

MSM: Men who have sex with men

There were eighteen genotypes found in 2009 only, most represented by one sample, and dates of collection are not recorded[16]. There were 23 common genotypes found in both 2009 and 2013, examples are given in Table 2.

**Table 2:** MLVA-*omp*A genotypes common between 2009[16] and 2012–13 studies.

MLVA-*omp*A genotype	Sex	Number of samples in 2009 study	Number of samples in 2012–13 study	Period Collected in 2012–13*
8.5.1-E	F	13	13	Sept 2012-Apr 2013
	M	0	7	
8.6.1-E	F	8	12	Sept 2012-Feb 2013
	M	0	7	
11.4.4-G	F	1	4	Sept 2012-Mar 2013
	M	0	0	
3.3.3-K	F	3	3	Oct 2012-Feb 2013
	M	0	1	
6.5.1-E	F	1	6	Sept 2012-Mar 2013
	M	0	2	

*date of collection was not recorded for 2009 study

## Discussion

We have conducted a detailed survey of the molecular epidemiology of *C*.*trachomatis* in the population of the UK city of Southampton between September 2012 and April 2013 by identification of MLVA-*omp*A genotypes among positive GUM clinic patients. This involved collection of demographic data to allow examination of possible trends in terms of indication of possible sexual networking within the city population. There were 380 positive samples obtained from 375 patients giving a prevalence of 12%. Of these 302 were fully genotyped by the MLVA-*omp*A scheme. The three most prevalent *omp*A genotypes were D, E and F, an observation which corresponds with the earlier study of Southampton[16] and other surveys[9, 14, 19]. The most common genotype 8.5.1-E (which constituted the founder group of Fig 1), was the same as the most common genotype identified amongst Southampton women in a detailed survey we conducted in 2009[16].

Geographical differences were apparent in relation to certain genotypes, for example the area of Southampton represented by postcode SO17. Of the two most common genotypes found in the study (8.5.1-E, n = 20; and 8.6.1-E, n = 19), only one example of the former was detected in SO17. Furthermore, the *omp*A distribution was observably different in SO17 compared to the other postcode-designated areas of Southampton (Fig 2). For example *omp*A genotype F is present at 0 to 11% in all areas except SO17, where it constituted 31% of the total genotypes in that ward. SO17 represents a unique electoral ward of the city in that it has a very diverse population, it is adjacent to the University of Southampton campus and consequently a quarter of its population of 14,831[24] comprises full-time students[25]. Therefore as this population has a large proportion of people with diverse nationalities, cultures, behaviours and likely networks owing to the nature of the university-related population, this may account of the differences seen between this and all the other Southampton postcode-defined areas.

We were able to identify genotypes among Southampton women in 2009 (the study was necessarily on samples from women due the fact we collected swabs (to allow tissue culture) and therefore no male samples, which were almost exclusively urine, were collectable)[16]. There appear to be two distinct groups of genotypes: those which persist over a long period (*i*.*e*. detected throughout the duration of the present study and often also found in Southampton in 2009[16]), and those which only seem to occur within a very short period (*i*.*e*. all or most samples collected with a relatively short time frame of three to four weeks). For instance the most common genotypes (8.5.1-E and 8.6.1-E), the former being the founder group of the MSpT (Fig 1), were the same as the most common genotypes identified in Southampton in 2009[16]. 8.5.1-E was sampled across a long period in the present study (September 2012 to April 2013) as well as its predominance in 2009, a similar case observable for 8.6.1-E. This suggests that these and other genotypes were well established within the sexually active population, indeed 8.5.1-E was frequently observed in a Japanese study[19] and in heterosexual men in China[26]. It is therefore likely 8.5.1-E is a stable genotype, widely distributed on a global scale. Conversely, there were notable genotypes which either occurred in 2009 or 2012/13 only, and as recorded in the latter study, they were typically obtained over relatively short periods, such as the four 8.3.4-D/UW-3/CX samples collected in a 2½ week period in 2012. This suggests either that such genotypes were relatively unstable in the population (which may mean they disappeared due to mutation to new genotypes) or that they circulated within a transient or very small sexual network.

Three notable *omp*A genotypes were identified: B, Ia and L2b. Genotype B is generally associated with patients who have trachoma rather than genital tract infections, furthermore the specific B genotype B3/IU-FQ279 is very rare in trachoma patients, although has been detected in adolescent women in the USA[27]. *Omp*A genotype Ia has previously been associated with black races[28], whereas all eight individuals in the present study were white British. *Omp*A genotype L2b is more commonly found in rectal samples of HIV positive MSM[13], therefore the infection of two heterosexual males in this study likely indicates a link to MSM sexual networks.

The current study was a prospective survey in which demographic data was collected together with the samples so that the provenance of the data associated with the inferred conclusions following analysis of the genotyping information could be assured. Previous studies using multi-locus typing schemes have used retrospective samples but included demographic and clinical data[3, 13, 29], therefore the present study contributes to the molecular epidemiological database for European countries. Our survey (2012–13) was conducted using exactly the same methodologies as in other studies conducted in Southampton[16] and Brighton[12], therefore comparison of data and population trends is reliable and not subject to discordance between different methodologies used in different studies. Furthermore, the MLVA-*omp*A genotyping scheme has been used in several overseas studies, allowing population trends to be compared on a global scale[7, 19, 26]. The terms of the ethical permission did not allow collection of patient data revealing contact information, hence partners could not be traced which would have enabled us to apply evidence of transmission chains and hence definition of specific sexual networks. Stability of the genetic types could not therefore be validated in a clinical setting.

Of the five individuals who supplied two samples on the same day, four had different MLVA-*omp*A genotypes in each of the pair of samples. Mixed infections were also recorded at a rate of 21% in a Tunisian study[30]. This and the fact that out of 302 genotyped samples 149 (49%) were of unique MLVA-*omp*A genotypes suggest a much higher discriminatory power than for example hrMLST[29]. Whilst this may run the risk of losing epidemiological links, the high discriminatory power of the MLVA-*omp*A system allows for a finer examination of small population groups. In some cases the different genotypes appear significantly diverse (e.g. 3.5.2-E and 3.4.3-J from a pharyngeal swab and a urine sample from one male). In other cases the genotypes are very similar according to sequence comparison, therefore could be a result of PCR proof-reading error, although previous assessment of the stability of the genotyping markers suggests such errors are unlikely[17]. Further studies would benefit from obtaining multiple samples from individuals to address the question of the level of co-infections among sexually active populations, as mixed infections have been recorded at 8.4% in of cases [31].

The most common genotype (8.5.1-E), as well as being the most numerous genotype found amongst Southampton women in 2009[16], was also the same as that found in a study of MSM in a similar period in another UK south coast town, Brighton[12]. This might indicate a “bridging” effect from a south coast heterosexual sexual network into an MSM network via bisexual activity. 8.5.1-E appeared to fall within an HIV negative network in Brighton, along with other “heterosexual *omp*A genotypes” such as D and F, indicating possible input from bisexual activity. The only genotypes which were common in MSM in both the present study and that in Brighton[12] (3.6.3-G, 3.5.3-J, 8.8.1-D/IC-CAL8 and 8.5.1-D/IC-CAL8) were only found in HIV negative MSM in Brighton, further suggesting bridging via bisexual activity as three of these genotypes were among the seven genotypes found in heterosexual males and females in the current study (S2 Table). Unlike an observation in China, the difference between genotypes circulating in the largely heterosexual population of this study and the MSM population of Brighton is subtle, in that the Southampton “heterosexual genotypes” correlate to the HIV negative sub-group of Brighton MSM, whereas the HIV positive sub-group differs, the latter containing more “MSM-like genotypes”[12, 26]. This may reflect a more fluid dynamic in network interactions in the UK compared to that in China, particularly in relation to MSM.

The ability to collect partner information during prospective sample and information gathering would help identify if these samples arose from the same sexual networks. However, the data presented can be used to infer sexual networks, including related behaviour and population dynamics. There are clearly genotypes which are well established as demonstrated temporally and geographically, and populations thus infected represent widespread sexual networks. These data strongly support the notion that interventions to reduce STIs in such networks should be broad national strategies, such as educational programmes. Conversely, there appear to be sexual networks which fall outside this definition. The application of the MLVA-*omp*A scheme showed that there are also sporadic (within a defined population and area) genotypes implying different sexual networks which might also be small and short-lived. It can only be implied that such networks represent different behaviour and culture, and therefore targeted surveillance and interventions may be required to identify such networks which do not act in the same way as widespread networks.

These data show that over a four year period (2009 to 2013), predominant circulating strains of *C*.*trachomatis* remain relatively stable.

## Supporting information

S1 Fig Minimum spanning tree of MLVA-*omp*A genotypes identified in Southampton in the present study (2012–13) and 2009[16]. Prominent clusters are coloured and are labelled with with the relevant MLVA-*omp*A designation.
(TIF)

S2 Fig Minimum spanning tree of MLVA-*omp*A genotypes identified in Southampton in the present study and 2009[16] showing distribution between the present study (2012–13) and 2009. Samples detected in 2009 are coloured red, specimens detected in the present study (2012–13) are coloured green (note this is exactly the same data as shown in S1 Fig but samples in clusters are colloured according to 20090or 2012–13).
(TIF)

S1 Table Distribution of *C*. *trachomatis* genotypes: MLVA relative to *omp*A. (XLSX)

S2 Table *C*. *trachomatis* MLVA-*omp*A genotypes in Southampton MSM. (XLSX)

S3 Table MLVA-*omp*A genotypes according to patient characteristics and geographical region in the city of Southampton. (XLSX)

S4 Table Comparison of MLVA-*omp*A genotypes detected in Southampton in the present study and in 2009[16], showing those detected in both studies. (XLSX)

S5 Table Minimal underlying data. (XLSX)
