# Supplementary figures and images for: Detailed molecular epidemiology of Chlamydia trachomatis in the population of Southampton attending the genitourinary medicine clinic in 2012-13 reveals the presence of long established genotypes and transitory sexual networks
Source: PLoS One. 2017 Sep 25;12(9):e0185059. doi: 10.1371/journal.pone.0185059 (PMC5612685; doi:10.1371/journal.pone.0185059)

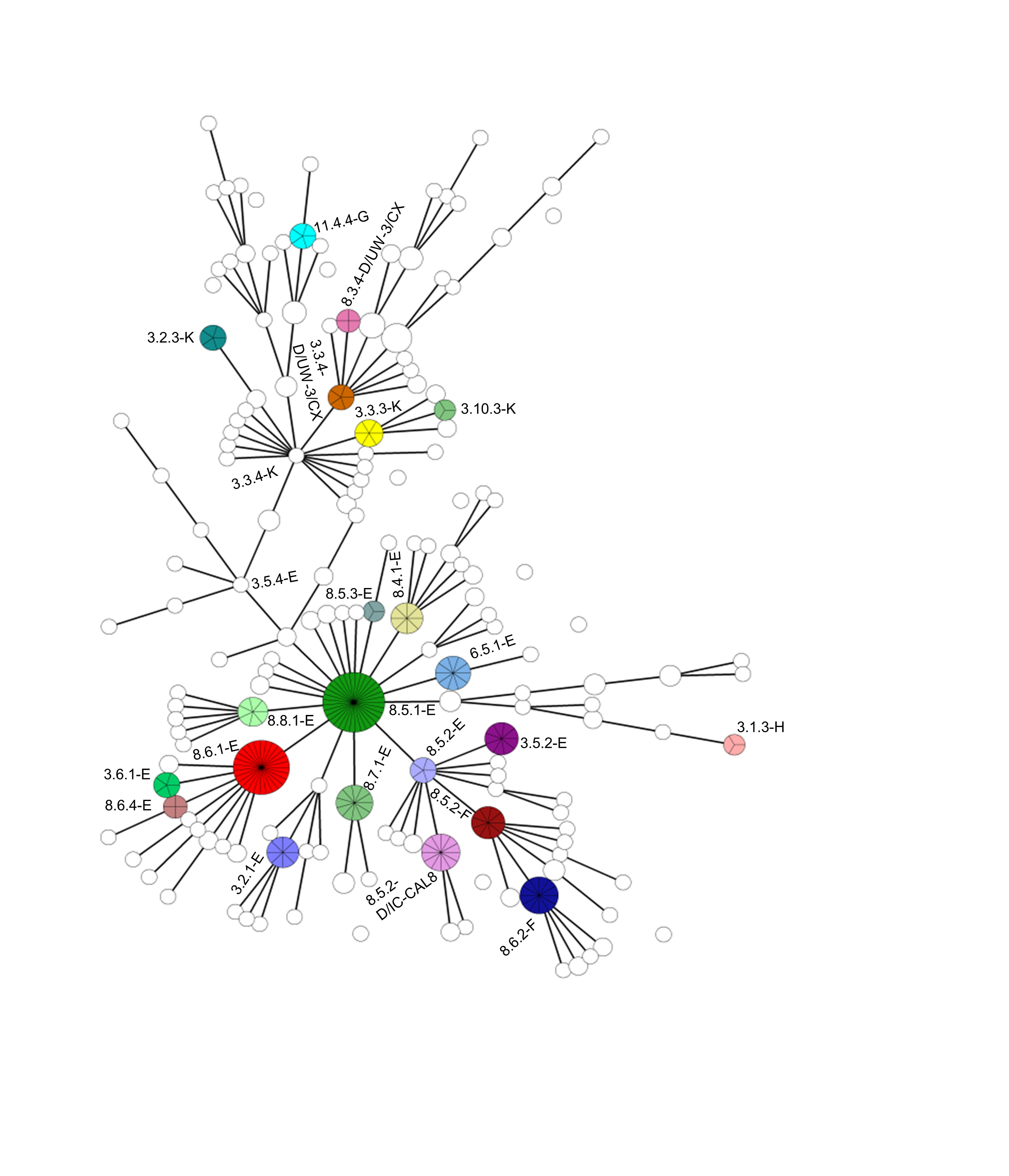

Supplement: S1 Fig — Prominent clusters are coloured and are labelled with with the relevant MLVA-ompA designation. (TIF) [file pone.0185059.s001.tif]

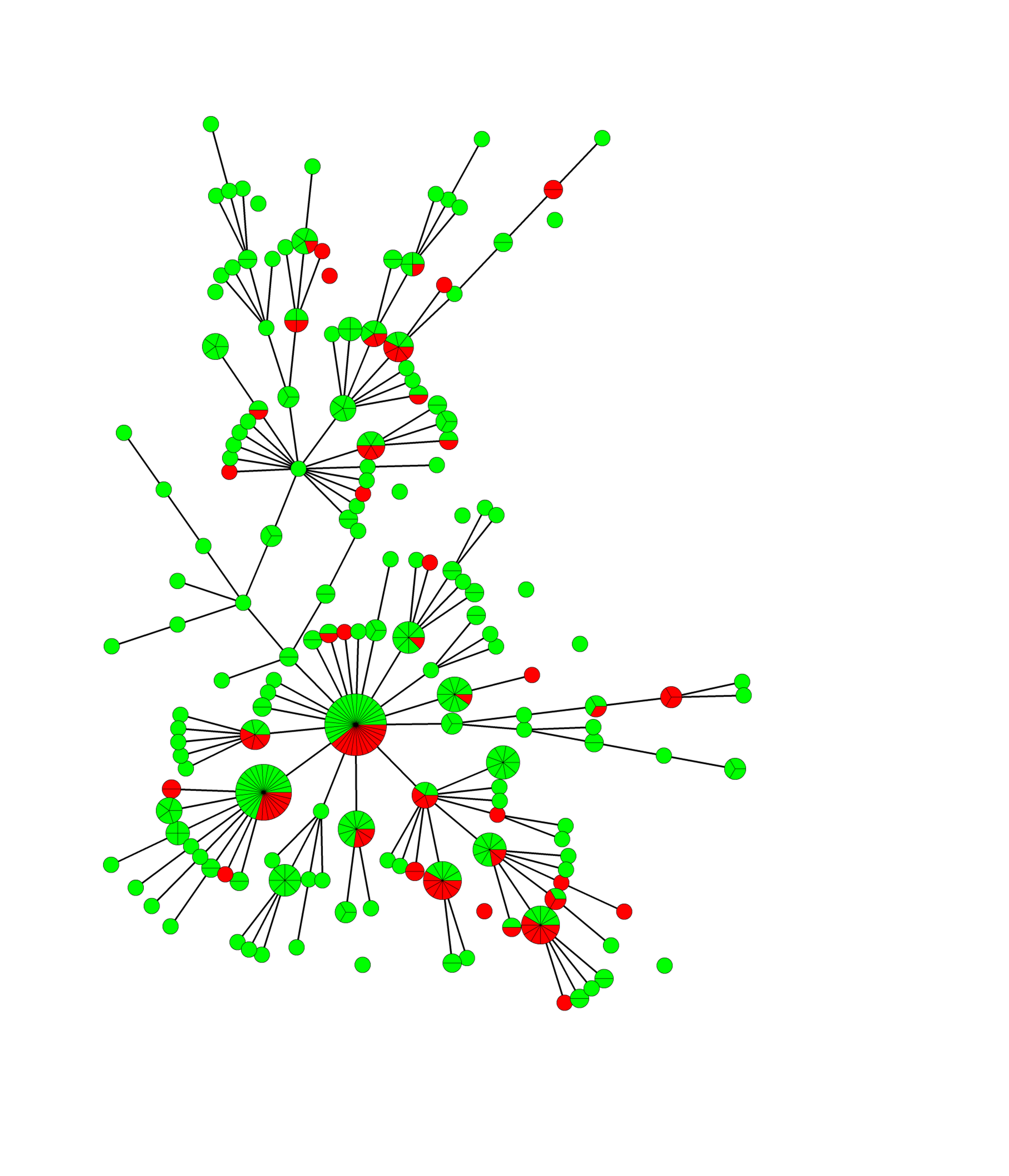

Supplement: S2 Fig — Samples detected in 2009 are coloured red, specimens detected in the present study (2012–13) are coloured green (note this is exactly the same data as shown in S1 Fig but samples in clusters are colloured according to 20090or 2012–13). (TIF) [file pone.0185059.s002.tif]
